# Protective effects of *Scutellariae Radix Carbonisata*-derived carbon dots on blood-heat and hemorrhage rats

**DOI:** 10.3389/fphar.2023.1118550

**Published:** 2023-08-10

**Authors:** Meiling Zhang, Jinjun Cheng, Juan Luo, Changxiang Li, Tingting Hou, Yan Zhao, Yaoxian Wang, Huihua Qu, Hui Kong

**Affiliations:** ^1^ School of Traditional Chinese Medicine, Beijing University of Chinese Medicine, Beijing, China; ^2^ Key Laboratory of Chinese Internal Medicine of the Ministry of Education, Dongzhimen Hospital Affiliated to Beijing University of Chinese Medicine, Beijing, China; ^3^ National Institute of TCM Constitution and Preventive Medicine, Beijing University of Chinese Medicine, Beijing, China; ^4^ Institute of Precision Medicine, Peking University Shenzhen Hospital, Shenzhen, China; ^5^ Merck & Co., Inc., Beijing, China; ^6^ Center of Scientific Experiment, Beijing University of Chinese Medicine, Beijing, China

**Keywords:** *Scutellariae Radix Carbonisata*-derived carbon dots (SRC-CDs), active ingredients, cooling blood and hemostatic effects, anti-inflammation, hemostasis

## Abstract

As the charcoal processing product of *Scutellariae Radix* (SR), SR Carbonisata (SRC) has been clinically used as a cooling blood and hemostatic agent for thousands of years. However, the underlying active ingredients and mechanism of SRC still remained unspecified. In this study, SRC derived carbon dots (SRC-CDs) were extracted and purified from the aqueous solution of SRC, followed by physicochemical property assessment by series of technologies. The cooling blood and hemostatic effects of SRC-CDs were further evaluated via a blood-heat and hemorrhage (BHH) rat model. Results showed that the diameters of obtained fluorescent SRC-CDs ranged from 5.0 nm to 10.0 nm and possessed functional group-rich surfaces. Additionally, the as-prepared SRC-CDs showed remarkable cooling blood and hemostasis effects in BHH model, mainly manifested by significant improvement of elevated rectal temperature, inflammatory cytokines (TNF-α, IL-6, and IL-1β) levels, as well as protein expressions of myD88 and NF-κB p65, abnormal coagulation parameters (elevated APTT and FIB), hemogram parameters (RBC, HGB, and HCT), and histopathological changes in lung and gastric tissues. This study, for the first time, demonstrated that SRC-CDs were the cooling blood and hemostatic active components of SRC, which could inhibit the release of inflammatory cytokines by regulating myD88/NF-κB signaling pathway, and activating the fibrin system and endogenous coagulation pathway. These results not only provide a new perspective for the study of active ingredients of carbonized herbs represented by SRC, but also lay an experimental foundation for the development of next-generation nanomedicines.

## Introduction

*Scutellariae Radix Carbonisata* (SRC), the charcoal product of *Scutellariae Radix* (SR, the dried root of *Scutellaria baicalensis* Georgi), has been used as cooling blood and hemostatic drug for more than one thousand years. The beneficial efficacy on treating various types of blood-heat and hemorrhage (BHH) symptoms such as vomiting blood, epistaxis and blood collapse (Huang et al., 2019) has been recorded by abundant ancient literatures and clinical cases, for example, the first recording on *Taiping Holy Prescriptions for Universal Relief* (978–992 AD, in China), *The Great Method of Processing* (1622 AD, in China) as well as modern clinic practices. Further pharmacological studies also verified the remarkable cooling blood and hemostatic effect of SRC via both BHH (Wang et al., 2011) and bleeding (Zhao et al., 2022) model, even a more enhanced cooling blood and hemostatic activity as compared with SR (Wang et al., 2011). However, in sharp to the definite efficacy, a gap on the study on active component and mechanism of SRC still existed, which need to be in-depth explored.

Different from other uncarbonized Chinese medicines, the results obtained from conventional analysis techniques (e.g., HPLC) were usually unsatisfactory during the process of analyzing the effective substance of carbonized Chinese medicines, leading to a dilemma on studying the effective substance of these carbonized Chinese medicines. In terms of SRC, the reason why SRC possessed more enhanced cooling blood and hemostatic effects than SR could not be explained on the base of the component analysis results obtained by HPLC method. For example, it has reported that the content of baicalin was decreased and the content of baicalein with little hemostatic activity (Kimura et al., 2001) was increased after carbonized processing (Wang et al., 2017), which was inconsistent with the more enhanced cooling blood and hemostasis effects of SRC. These research results suggest that we should adopt a new perspective and strategy to study the effective substance of SRC.

Previous studies have shown that components such as lignin in wood (e.g., SR) was pyrolytic and formed into coke during the high-temperature carbonization process (Cao et al., 2012), followed by a significant increase in the “carbon” content of charring products (e.g., SRC). Combined with the enhanced hemostatic activity of carbonized production, we speculated that the newly produced “carbon” was the key substance of SRC for cooling blood and hemostasis effect. However, what exactly the “carbon” is has not been deeply explored and clarified (Huang et al., 2013).

Inspired by previous reports (Yan et al., 2017; Zhang et al., 2018) that carbon dots (CDs) with multiple biological activities could be synthesized from herbs after high temperature carbonization treatment, we proposed that the newly produced “carbon” of SRC might be a new type of CDs, which is closely related to its cooling blood and hemostatic biological activities. Following this lead, novel CDs was found by isolating and purifing the aqueous solution of SRC via dialysis method in previous study, which were named as SRC-derived CDs (SRC-CDs). Further study (Kong et al., 2021) showed that the obtained SRC-CDs exerted anti-inflammatory effects, mainly manifesting as reducing in inflammatory cytokines levels in C48/80-induced RBL-2H3 cell model.

According to the previous report (Liu et al., 2023), the progression of BHH is usually accompanied by bleeding as well as inflammation. Herein, we hypothesized that SRC-CDs was the cooling blood and hemostatic component of SRC and investigated its activity using a rat BHH model for the first time. Then we explored whether the underlying mechanism was partly mediated through the modulation of myD88/NF-κB signaling pathway and coagulation system.

## Materials and methods

### Materials and reagents

SR (Batch number: 211013005) was purchased from Beijing Qiancao Herbal Pieces Co., Ltd. (Beijing, China) and SRC was prepared in our laboratory. Yunnan Baiyao Capsules were brought from Yunnan Baiyao Group Co., Ltd. (Kunming, China). Dialysis membranes with a molecular weight cut-off of 1,000 Da were provided by Beijing Ruida Henghui Technology Development Co., Ltd., (Beijing, China). Sodium citrate (3.8%), paraformaldehyde (4%), sodium carboxymethylcellulose and other HPLC- or analytical-grade chemical reagents were obtained from Sinopharm Chemical Reagents Co., Ltd. (Beijing, China). Dry yeast was purchased from Hubei Angel Yeast Co., Ltd. (Wuhan, China). The ELISA kits for measuring tumor necrosis factor-α (TNF-α), interleukin 6 (IL-6), and interleukin 1β (IL-1β) were purchased from Proteintech Group, Inc. (Wuhan, China) and Abcam (Cambridge, United Kingdom), respectively. Primary antibodies against mouse myD88 (67969-1-AP), mouse GAPDH (60004-1-lg), mouse β-actin (66009-1-lg) and rabbit NF-κB p65 (10745-1-AP) were purchased from Proteintech Group, Inc. (Wuhan, China). Additionally, deionized water (DW) was used throughout the experiment.

### Animals

Kunming mice (weight: 28.0 ± 2.0 g, male) and Sprague-Dawley rats (weight: 200.0 ± 10.0 g, male) were provided by Si Peifu Biotechnology Co., Ltd. All animals were kept in the same experimental environment for 3 days before the experiment, with an ambient temperature of (24.0 ± 1.0) °C and relative humidity of 55%–65%. All experimental animals were provided free access to unlimited amounts of water and food, and were kept under a 12 h light and dark cycle.

### Preparation of SRC, SRC-CDs and the solution outside the dialysis membrane of SRC (SRC-OD)

**FIGURE 1:**
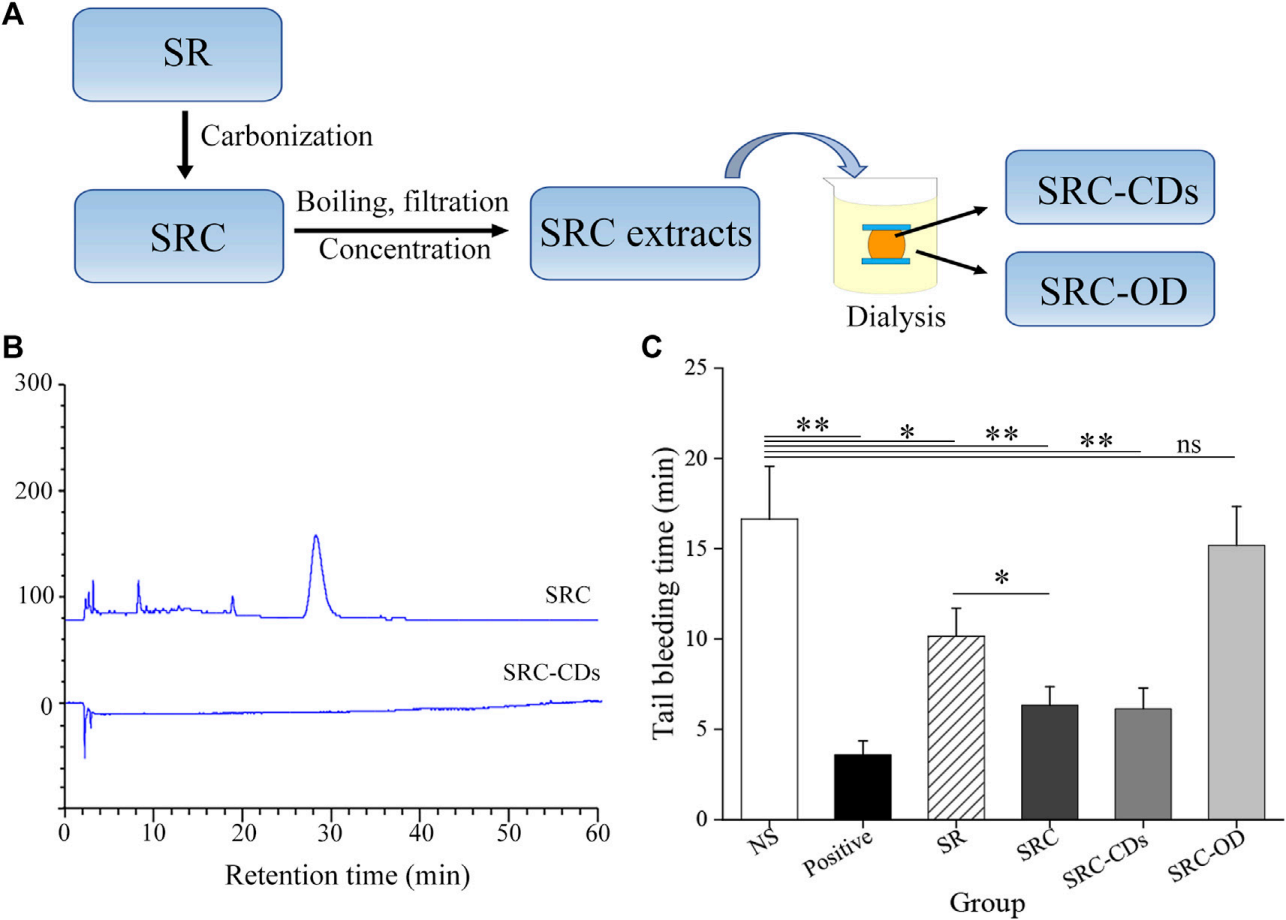
**(A)** Flow diagram of preparing process of *Scutellariae Radix Carbonisata—*derived carbon dots (SRC-CDs) and the solution outside the dialysis membrane of SRC (SRC-OD). **(B)** HPLC fingerprint of SRC and SRC-CDs. **(C)** Tail bleeding time of mice (*n* = 6 per group) treated with normal saline (NS), Yunnan Baiyao (positive), *Scutellariae Radix* (SR), SRC, SRC-CDs and SRC-OD. Data are represented as mean ± SD. *p < 0.05 vs. NS group or SR group and **p < 0.01 vs. NS group.

SRC was prepared in our laboratory by a calcination method with under modified condition (Zhang et al., 2021). Briefly, SR was calcined at 350°C for 30 min using a muffle furnace (TL0612, Beijing, China) as the carbonization equipment. The obtained SRC were crushed and extracted twice with DW at 100°C, followed by filtrating through a 0.22 μm microfiltration membrane to remove the macromolecular weight products. Yellowish-brown solution was collected and concentrated to obtain SRC extracts. After that, the SRC-CDs inside the dialysis membrane (MWCO = 1000) and the solution outside the dialysis membrane (SRC-OD) were separately collected by dialysis against DW for 5 d, centrifuged at 11,000 rpm for 30 min, and stored at 4°C until use. The preparation flow diagram was illustrated in Figure 1A.

### Fingerprint of SRC and SRC-CDs by HPLC analysis

A comparative analysis of chemical composition between SRC and SRC-CDs was carried out using an Agilent 1,260 series HPLC instrument (Agilent, Waldbronn, Germany). The prepared aqueous solution of SRC and SRC-CDs were filtered through a 0.22 µm cellulose membrane and analyzed under modified conditions. Specifically, a Reliash-C18 column (250 mm × 4.6 mm, 5 μm, Orochem, Illinois, United States) was applied to separate SRC and obtained SRC-CDs both, with a column temperature of 35°C. The mobile phase was 0.05% phosphoric acid (A) and methanol (B), with a modified gradient program performing as following: 0–10 min, 10%–15% B; 10–20 min, 15%–60% B; 20–50 min, 60%–80% B; 50–60 min, 80%–85% B. The flow rate was 1 mL/min and the detection wavelength set as 254 nm.

### Characterization of SRC-CDs

The morphology of SRC-CDs was obtained by a JEN-1230 high-resolution transmission electron microscopy (HRTEM, JEOL, Tokyo, Japan). The optical properties of SRC-CDs were analyzed by ultraviolet visible (UV-Vis) spectrophotometer (Cecil, Cambridge, United Kingdom), fluorescence spectrophotometer (F-4500, Hitachi, Tokyo, Japan), and Fourier transform infrared spectrometer (FTIR, Thermo, Massachusetts, United States). The surface composition and elemental analyses of the as-prepared SRC-CDs were performed by X-ray photoelectron spectrometer (XPS, ESCALAB 250Xi, Fremont, CA, United States) with a mono X-ray source Al Kl excitation (1,486.6 eV). X-ray diffractometer (XRD, Ultima IV, Tokyo, Japan) patterns were obtained using an X-ray diffractometer with Cu Kalpha radiation (λ = 1.5418 Å).

Additionally, the quantum yield (QY) of the prepared SRC-CDs was determined as previously described using quinine sulfate (% QY = 54, in 0.1 M H_2_SO_4_ solution) as a referenced sample (Liu et al., 2020). The QY of SRC-CDs was calculated according to the following equation.

$$Q_{CDs}=Q_{R}\times \frac{I_{CDs}}{I_{R}}\times \frac{A_{R}}{A_{CDs}}\times \frac{\eta_{CDs}^{2}}{\eta_{R}^{2}}$$

In the above equation, “Q” represents the QY and “η” represents the refractive index of the solvent. “Ι” and “A” are the integrated areas of the emission spectrum and absorbance obtained at 409 nm, respectively. The subscript “CDs” denotes the obtained SRC-CDs while “R” refers to the standard. ηCDs/ηR = 1 in aqueous solution. To minimize reabsorption interference, the absorbance of “CDs” and “R” were less than 0.05.

### Hemostasis study of SR, SRC, SRC-CDs and SRC-OD in mice tail bleeding model

Mice were randomly divided into 6 groups (*n* = 6) and administered by gavage as follows: 1) control group (mice treated with normal saline, NS); 2) positive group (mice treated with Yunnan Baiyao, 250 mg/kg); 3) SR group; 4) SRC group; 5) SRC-CDs group; 6) SRC-OD group. The doses of SR and SRC were both 500 mg/kg, while the doses of SRC-CDs and SRC-OD were equivalent to 500 mg/kg SRC. The mice tail bleeding model was established as previously described (Cheng J. et al., 2019). Briefly, tails of mice with a diameter of approximately 1.08–1.12 mm were transacted using a sterile scalpel and then immediately placed on a filter paper. The bleeding time was monitored at an interval of 30 s until hemorrhage was completely ceased, where hemostasis maintained for 30 min could be define as hemostatic endpoint (Zhu et al., 2015). All animals were euthanized at the end of the experiment using the cervical dislocation method.

### Cooling blood and hemostasis study of SRC-CDs in a rat BHH model

Sixty SD rats were randomly divided into 6 groups with 10 rats each: 1) Control group; 2) Model group; 3) Positive group (rats treated with Yunnan Baiyao, 250 mg/kg); 4) SRC-CDs at high dose group (25.00 mg/kg); 5) SRC-CDs at medium dose group (12.50 mg/kg); and 6) SRC-CDs at low dose group (6.25 mg/kg) (rats treated with different doses of SRC-CDs). Animals were administrated with 10 mL/kg of each drug by gavage once a day for 7 consecutive days. To establish BHH model (Cheng P. et al., 2019), rats in model, positive and SRC-CDs (high/medium/low) groups were subcutaneously injected with 20% dry yeast suspension (mass fraction) on the back (10 mL/kg), followed by 1 mL anhydrous ethanol 5 h post dry yeast intervention. The control group was simultaneously given an equal volume of DW. 1 h later, rats of each group were given the corresponding drugs by gavage. Then rectal temperature of rats was measured at 0, 2, 4, 6, and 8 h after the injection of 20% dry yeast suspension. 1 h after the last administration on the 8th day, rats in each group were anesthetized with 10% chloral hydrate. Blood and tissues samples were collected for further analysis.

### Histological examination of lung and gastric tissues

The lung and gastric tissues of rats were excised, rinsed with saline and then observed visually for hemorrhage phenomenon. Then the specimens were preserved with 4% paraformaldehyde solution and paraffin-embedded. After staining with hematoxylin and eosin (HE), pathological changes of the tissue sections were observed under a light microscope.

### Determination of coagulation parameters

Whole blood was collected in plastic tubes containing 3.8% sodium citrate (blood: sodium citrate 9:1, v/v), followed by centrifugation at 750 × g for 15 min to obtain plasma. Coagulation parameters including plasma prothrombin time (PT), activated partial thromboplastin time (APTT), thrombin time (TT) and fibrinogen (FIB) levels were determined using a CA-500 automatic coagulation analyzer (Sysmex, Japan). The test was completed within 4 h.

### Determination of hematologic parameters

Red blood cell count (RBC), hemoglobin concentration (HGB) and red blood cell specific volume (HCT) in EDTA-K_2_ anticoagulated blood samples were determined using a XS-800i blood cell automatic analysis device (Sysmex, Japan).

### Determination of inflammatory cytokines in plasma and tissues

Commercially available ELISA kits were used to detect TNF-α, IL-1β and IL-6 levels in plasma, lung and gastric tissues, according to the manufacturer’s instructions.

### Western blot analysis

Total protein of lung and gastric tissues were extracted using cold RIPA buffer with 1% proteinase inhibitor, and the concentrations of it were quantified applying a Bis-creatine (BCA) kit. Then equal amounts of proteins from both lung and gastric tissues were separated by SDS-PAGE electrophoresis, followed by being transferred to NC membranes after blocking with 5% skimmed milk for 1.5 h at room temperature. Proteins were then probed with corresponding primary antibodies against myD88 and NF-κB at 4°C overnight, followed by incubation with HRP-conjugated secondary antibodies for 1 h at room temperature. After cleaning with TBST, the target protein in NC membranes was visualized by an automatic chemiluminescence image analysis system (5,200, Shanghai, China) using enhanced chemiluminescence method.

### Statistical analysis

The statistical analysis was conducted using SPSS software (version 25.0, United States). Multiple comparisons were performed using one-way ANOVA followed by least significant difference (LSD) tests. Normally-distributed variables and those with homogeneous variances were expressed as mean ± standard deviation (SD). A threshold value of *p* < 0.05 indicates statistically significant differences between groups.

## Results

### Comparative analysis of chemical ingredients in SRC and SRC-CDs

The compositional differences between SRC and SRC-CDs were analyzed by HPLC method. As indicated in Figure 1B, multiple peaks could be observed in the fingerprint spectra of SRC, in marked contrast to no peaks in the HPLC fingerprint of SRC-CDs solution. This result suggested that small moleculars in SRC-CDs aqueous solution have been successfully separated by dialysis method.

### Exploring the hemostatic active ingredients of SRC

The hemostatic activity of SR and SRC was compared using the mouse tail bleeding model. The result showed that the tail bleeding time was significantly shorter in SRC-treated animals than in SR-treated controls (*p* < 0.05, Figure 1C), demonstrating that charcoal powder concoction enhanced the hemostatic activity of SR. To further investigate whether SRC-CDs are the hemostatic active components of SRC, we compared the hemostatic activity of SRC, SRC-CDs and SRC-OD. In contrast to the longer bleeding time in mice after SRC-OD (*p* > 0.05 vs. NS-treated mice) intervention, both SRC and SRC-CDs groups exerted conspicuous effects on shortening the bleeding time of tail bleeding mice (*p* < 0.01 vs. NS-treated mice). Notably, comparative hemostatic activity could be observed between SRC and SRC-CDs groups. These results demonstrated that SRC-CDs were the main hemostatic active componet of SRC.

### Characterization of SRC-CDs

#### Morphology characterization

**FIGURE 2:**
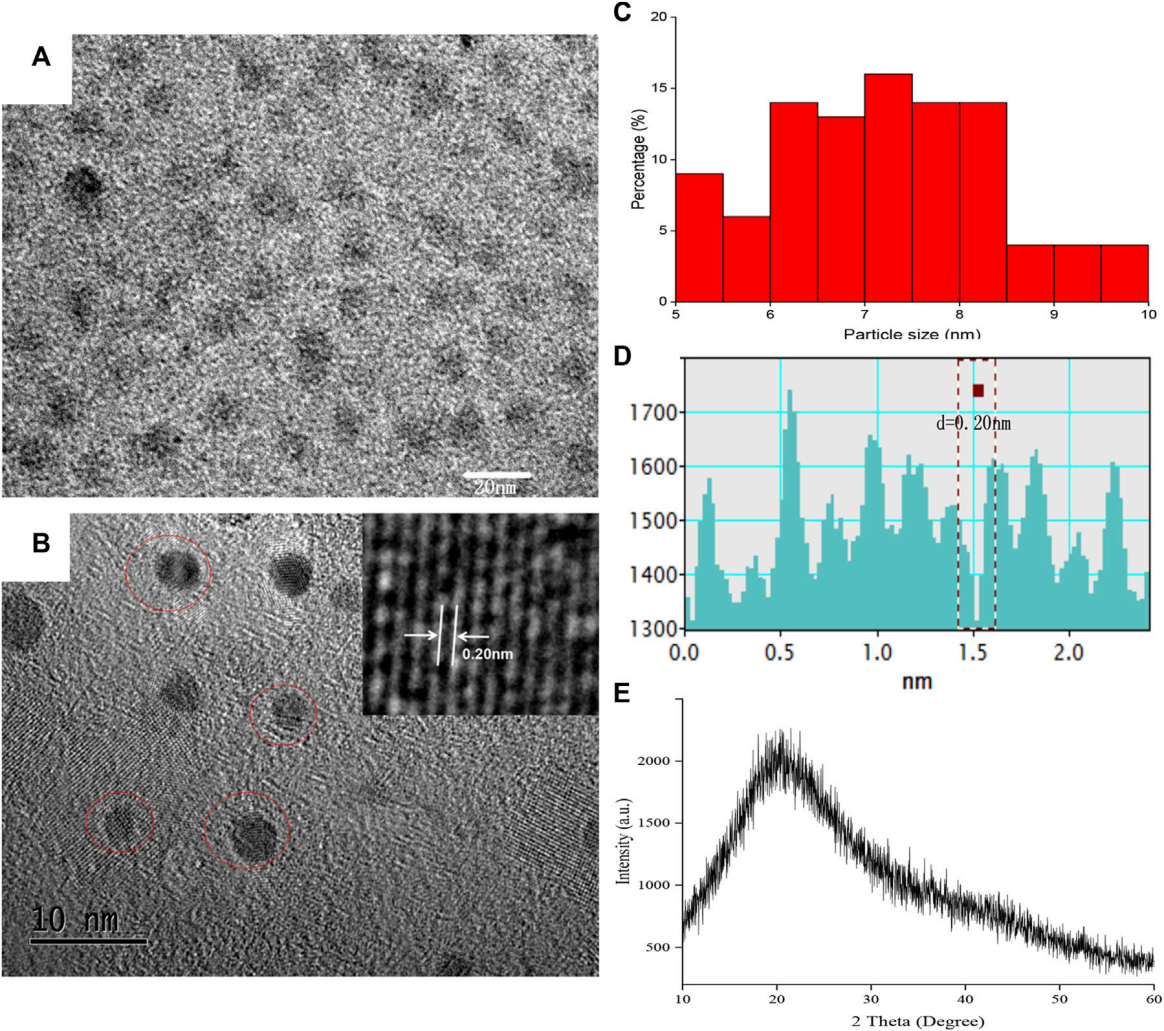
The morphology characterization of *Scutellariae Radix Carbonisata—*derived carbon dots (SRC-CDs). **(A,B)** High-resolution transmission electron microscopy (HRTEM) images. Inset: magnification figure. **(C)** Histogram depicting particle size distribution. **(D)** Line profiles of the corresponding HRTEM images and **(E)** X-ray diffraction pattern (XRD) of SRC-CDs.

The HRTEM image showed that as-prepared SRC-CDs were subspherical particles (Figures 2A, B) with size distribution ranging from 5.0 nm to 10.0 nm (Figure 1C) and a lattice spacing of 0.20 nm (Figures 2B–D). Additionally, a wide diffraction peak could be observed in XRD pattern (Figure 2E), revealing the highly amorphous nature of the obtained SRC-CDs (Liu et al., 2012).

#### Optical characterization

**FIGURE 3:**
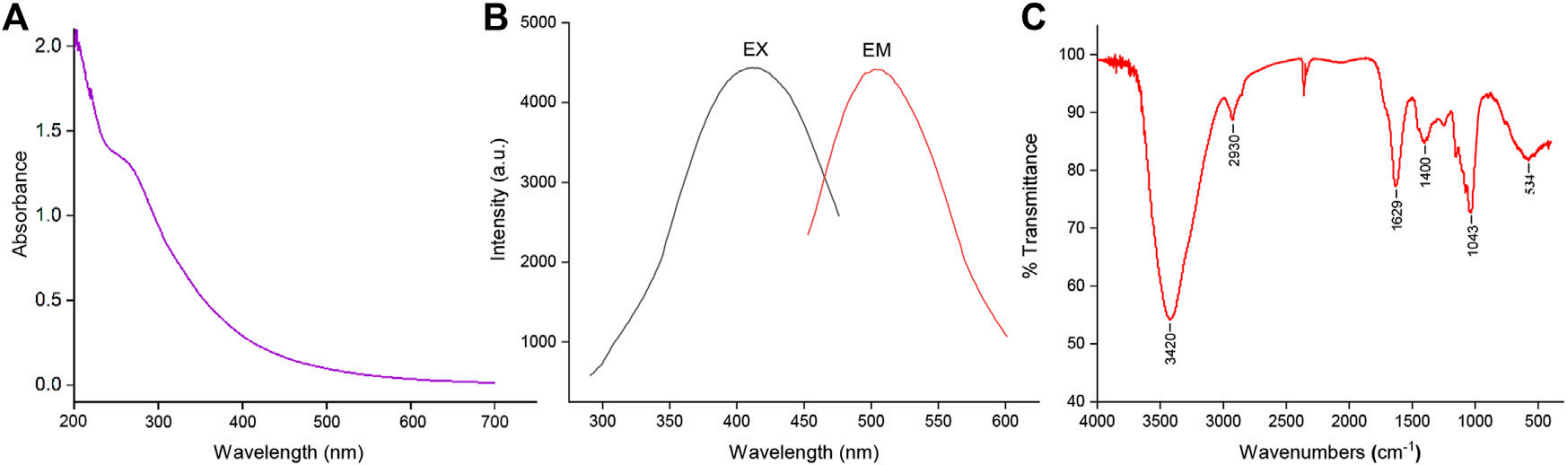
The optical characterization of *Scutellariae Radix Carbonisata—*derived carbon dots (SRC-CDs). **(A)** Ultraviolet-visible spectrum (UV-Vis), **(B)** fluorescence spectrum and **(C)** Fourier transform infrared spectrum (FTIR).

The optical properties of SRC-CDs were characterized using UV-vis and fluorescence spectroscopy. As indicated in Figure 3A, a weak absorption peak of SRC-CDs could be observed around 270 nm, corresponding to the π–π* transition of the conjugated C=C bonds and aromatic sp^2^ domains (Wang et al., 2016). The fluorescence properties of SRC-CDs shown in Figure 3B exhibited the maximum emission and excitation wavelength around 503 nm and 409 nm, respectively. Additionally, the QY of SRC-CDs was calculated to be 3.26% using quinine sulfate as a standard.

#### Surface functional groups properties

**FIGURE 4:**
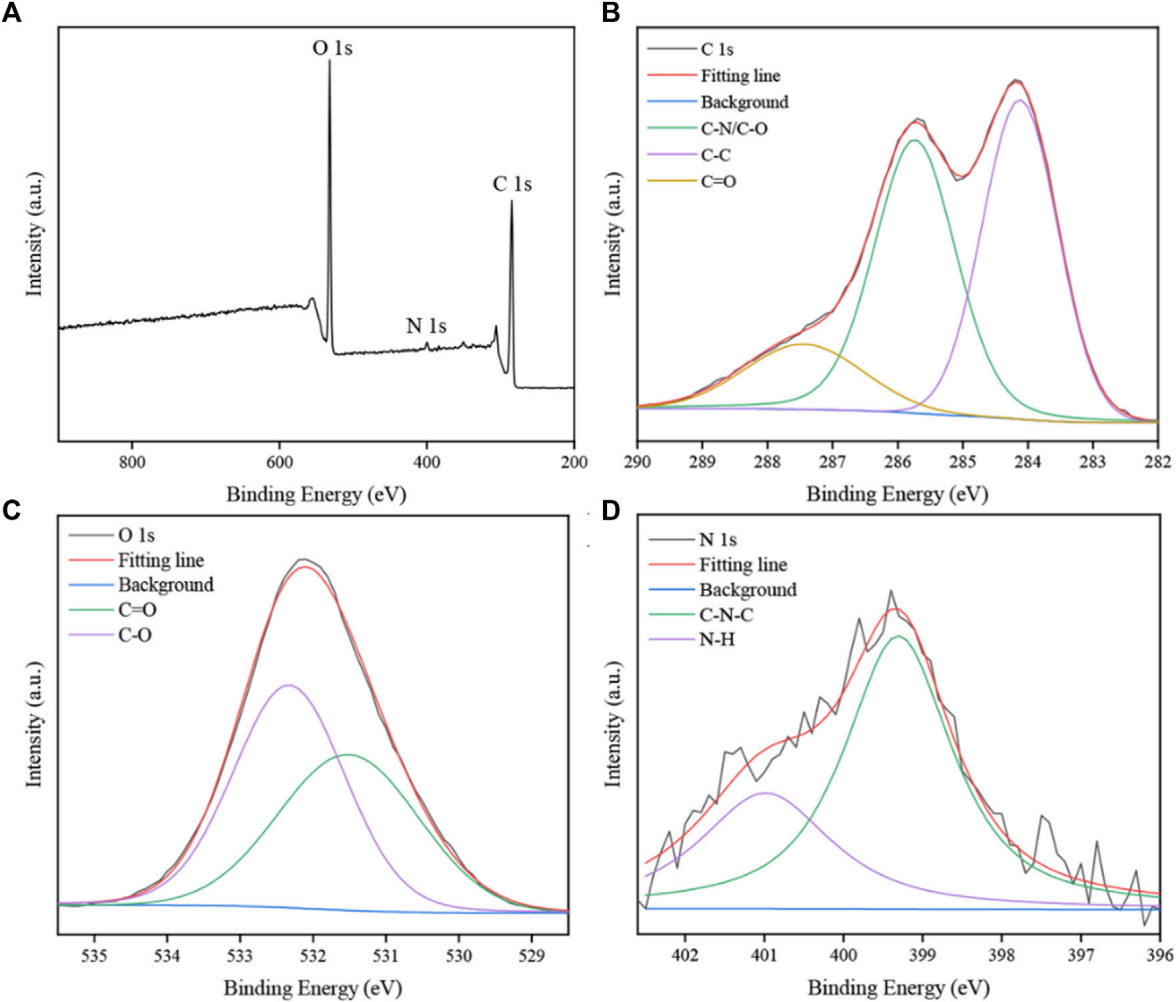
The X-ray photoelectron spectroscopy (XPS) spectra of *Scutellariae Radix Carbonisata—*derived carbon dots (SRC-CDs). **(A)** Full survey spectrum. **(B)** C 1s spectrum, **(C)** O 1s spectrum and **(D)** N 1s spectrum.

The FTIR spectra of obtained SRC-CDs identified characteristic peaks at 3,420 cm^−1^, 2,930 cm^−1^, 1,629 cm^−1^, 1,043 cm^−1^, and 534 cm^−1^, as shown in Figure 3C. Among them, the broad absorption at 3,420 cm^−1^ was attributed to the absorption bands of O-H and N-H stretching vibrations, and the peak at 2,930 cm^−1^ was ascribed to the C–H stretching vibrations (Zhang et al., 2019). The weak peak at 1,400 cm^−1^ was attributed to C-N bonds (Wang et al., 2018). The peaks at 1,629 cm^−1^ and 1,043 cm^−1^ were identified as C=O and C-O-C bonds, respectively. The above results confirmed the existence of a series of active functional groups such as carboxyl, amino, amidogen, hydroxyl on the surface of SRC-CDs (Zhao et al., 2017). The elemental ratio and surface composition of as-prepared SRC-CDs was further analysized by XPS, which has been exhibited in Figure 4. Three typical peaks associated with elements C, N and O could be observed in XPS survey spectrum (Figure 4A), with relative contents of 65.04%, 32.29%, and 2.16%, respectively. The C1s high-resolution XPS spectra (Figure 4B) confirmed the existence of C-N/C-O (285.7 eV), C-C/C=C (284.1 eV) and C=O (287.7 eV). Two distinct peaks at 531.5 eV and 532.3 eV in high-resolution O 1s spectra (Figure 4C) could be assigned into C-O and C=O, respectively. In addition, the N1s spectra relvealed two peaks at 399.3 eV and 400.99 eV, corresponding to C-N-C and N-H, respectively (Figure 4D). These results (Sun et al., 2017; Fu et al., 2019) were consistent with the FTIR analysis and demonstrated the presence of above-mentioned active functional groups on the surface of obtained SRC-CDs. The characterization parameters of SRC-CDs were summerized in Table 1.

**TABLE 1:** The characterization parameters of obtained SRC-CDs.

Characterization	Parameters
Particle size distribution (nm)	5.00 ± 10.00
Lattice structure (nm)	0.20
Ultraviolet absorption (nm)	270
Maximum excitation/emission wavelength (nm)	409/503
Element ratio	C: 65.04%
	O: 32.29%
	N: 2.16%
Quantum yield	3.26%
Chemical bond	C-N/C-O, C-C/C=C, C=O, N-H, O-H, etc

### Effects of SRC-CDs on rectal temperature of rats

The rat rectal temperature at time intervals of 0, 2, 4, 6, 8 h before and after injection with 20% dry yeast solution was measured and recorded in Table 2. Compared with the control group, the rectal temperature of rats injected with dry yeast only started to increase significantly at 2 h post injection (*p* < 0.01) and maintained at a high level during 4 h–8 h (*p* < 0.01), indicating that the rat BHH model was successfully established. Compared with the model group, the rectal temperature of rats was reduced to different degrees after treated with different doses of SRC-CDs. Particularly, at 2 h, both high and low doses of SRC-CDs significantly inhibited elevated rectal temperature of rats induced by dry yeast (*p* < 0.05). Additionally, the time (8 h) to reach the maximum rectal temperature in rats from SRC-CDs groups was significantly delayed compared with the time (4 h) to reach the maximum rectal temperature in the model rats, demonstrating the cooling-blood effects of SRC-CDs to some extent. However, rectal temperature of rats in positive drug group was not significantly different from that of the model group at different time points.

**TABLE 2:** Effects of SRC-CDs on rectal temperature in blood heat and hemorrhage rats ( $\bar{x}$ ± *SD*, *n* = 10).

Groups	Time					
	0 h	2 h	4 h	6 h		8 h
Control	37.31 ± 0.15	37.33 ± 0.21	37.24 ± 0.15	37.31 ± 0.14	37.35 ± 0.15	
Model	37.37 ± 0.35	38.37 ± 0.45^**^	39.04 ± 0.3^**^	39.13 ± 0.27^**^	39.07 ± 0.25^**^	
Positive	37.36 ± 0.4	38.05 ± 0.43	38.53 ± 0.36	39.05 ± 0.3	39.11 ± 0.28	
SRC-CDs 6.25 mg/kg	36.65 ± 0.52	37.76 ± 0.22^#^	38.88 ± 0.35	39.06 ± 0.29	39.18 ± 0.30	
SRC-CDs 12.50 mg/kg	36.94 ± 0.46	38.6 ± 0.50	38.71 ± 0.43	39.04 ± 0.31	39.27 ± 0.24	
SRC-CDs 25.00 mg/kg	37.18 ± 0.29	37.95 ± 0.38^#^	38.21 ± 0.55	38.99 ± 0.34	39.18 ± 0.31	

Note: ^*^ *p* < 0.05 and ^**^ *p* < 0.01 compared with control group; ^#^ *p* < 0.05 compared with model group.

### Histopathological analysis of lung and gastric tissues

**FIGURE 5:**
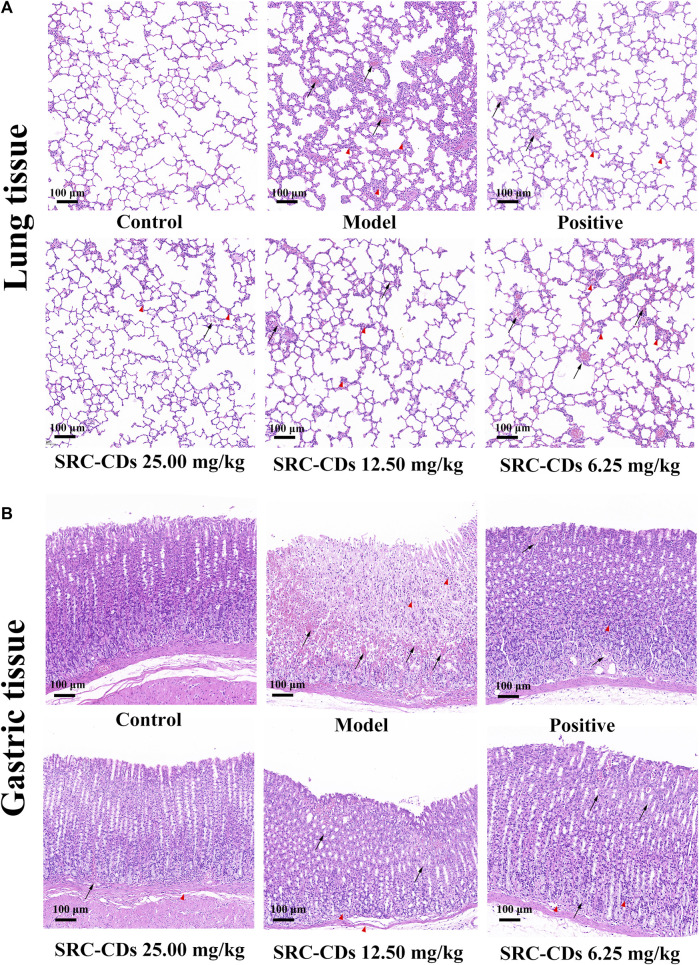
Histopathology of **(A)** lung tissues and **(B)** gastric tissues (×100). Sprague Dawley rats were assigned into six groups (*n* = 10): control (NS), model, positive and *Scutellariae Radix Carbonisata—*derived carbon dots (SRC-CDs) at high- (25.00 mg/kg), medium- (12.50 mg/kg) and low (6.25 mg/kg) doses groups. The arrows represents local bleeding point, while “△(red)” represents inflammatory cells infiltration.

As shown in Figure 5A, the rat lung tissues in NS group was pink in color with normal morphology and clear alveolar structure. In contrast, the lung histopathological changes in rats with BHH were obvious, mainly manifesting as more hemorrhagic spots, hyperplasia of fibrous tissue, local inflammatory cell infiltration, and partial destruction of alveolar structure. After the animals were orally administered with three doses of SRC-CDs, all of the above lung lesions were better relieved. Similar phenomenons were observed in the positive group. The gastric histopathological characteristics of rats in each group were further analyzed to investigate the protective effect of SRC-CDs against gastric injury caused by the combined application of dry yeast and anhydrous ethanol. It could be clearly seen that the control rats had normal gastric wall structure, smooth and flat gastric mucosa, and neatly arranged glandular cells (Figure 5B). After modelling, the gastric tissues of hemorrhagic rats were severely damaged, mainly manifested by severe intervillous hemorrhage, submucosal inflammatory cells infiltration and disorganized glandular cells arrangement. These pathological changes were significantly reversed by the intervention with positive drugs and SRC-CDs at high-, medium- and low doses. Compared with the normal group, the gastric tissue structure of the animals in the high dose groups was basically normal, except for slight gastric damage and bleeding in the middle and low dose group. The above results indicated that SRC-CDs had obvious protective effects on lung and gastric tissues damage in BHH syndrome rats.

### Effects of SRC-CDs on plasma coagulation parameters

**FIGURE 6:**
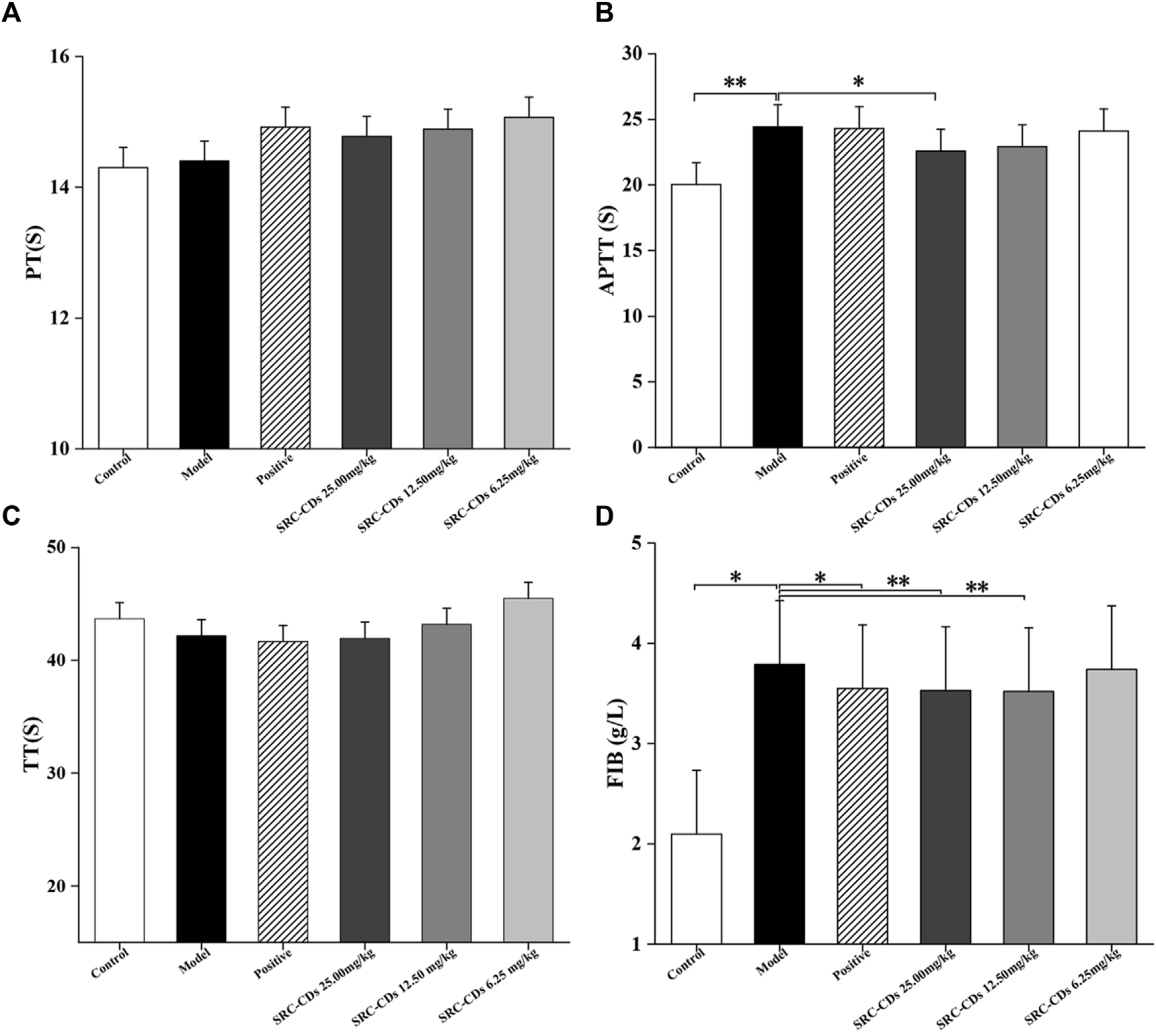
Effects of *Scutellariae Radix Carbonisata—*derived carbon dots (SRC-CDs) on coagulation parameters. **(A)** Prothrombin time (PT), **(B)** activated partial thromboplastin time (APTT), **(C)** thrombin (TT) and **(D)** fibrinogen (FIB). Sprague Dawley rats were assigned into six groups (*n* = 10): control, model, positive and SRC-CDs at high- (25.00 mg/kg), medium- (12.50 mg/kg) and low (6.25 mg/kg) doses. *p < 0.05 and **p < 0.01 vs. model group or control group.

To investigate the anticoagulant effect of SRC-CDs on BHH model, coagulation parameters (PT, APTT, TT, and FIB) of rats in different groups were measured and analysed. An elevated APTT (Figure 6B, *p* < 0.01 vs. control group) and FIB value (Figure 6D, *p* < 0.05 vs. control group) in model group suggested the successful establishment of BHH model, which was consistent with previous reports (Qi et al., 2019). Notably, intervention with SRC-CDs significantly improved elevated APTT (high dose: *p* < 0.05) and FIB (high- and medium doses: *p* < 0.01) in rat plasma, suggesting that the potential mechanism by which the as-prepared CDs exert hemostatic effects was partly related to endogenous coagulation pathways and activation of fibrin system (Zhao et al., 2017; Luo et al., 2018). Similar observations could be found in the plasma FIB of rats treated with SRC-CDs at low dose. Additionally, although there was no significant difference in the effects of medium- and low doses of SRC-CDs on APTT, a positive tendency was observed in both doses. In terms of PT (Figure 6A) and TT (Figure 6C) measurements, there were was no statistic differences between all groups, tentatively suggesting that the hemostatic/hemorrhage effects of SRC-CDs/dry yeast was not associated with exogenous coagulation pathway (Luo et al., 2018). Oral administration of Yunnan Baiyao only reduced plasma APTT and FIB values in blood heat and hemorrhage rats, but had little effects on the remaining indicators (PT and TT).

### Effects of SRC-CDs on hematologic parameters

As seen in Table 3, there was a trend of elevated RBC, HGB and HCT in the model group rats compared with the control group, but the difference was not statistically significant. Notably, high- (*p* < 0.05) and low (*p* < 0.05) doses of SRC-CDs intervention significantly ameliorated the elevated HCT in BHH rats. Also, a positive trend of lower RBC and HGB was observed after the intervention of all three doses of SRC-CDs in model rats, suggesting that the obtained CDs may have the potential to reduce whole blood viscosity and improve blood flow.

**TABLE 3:** Effects of SRC-CDs on hemogram parameters in blood heat and hemorrhage rats ( $\bar{x}$ ± *SD*, *n* = 10).

Groups	RBC/10^12^/L	HGB/g·L^−1^	HCT
Control	5.21 ± 0.01	110.50 ± 1.50	37.75 ± 0.45
Model	5.95 ± 0.65	122.67 ± 13.89	39.9 ± 4.96
Positive	5.91 ± 0.28	119.25 ± 5.97	39.45 ± 1.72
SRC-CDs 6.25 mg/kg	5.39 ± 0.81	109.70 ± 14.91	35.12 ± 1.73^#^
SRC-CDs 12.50 mg/kg	5.79 ± 0.63	119.37 ± 12.28	38.05 ± 3.47
SRC-CDs 25.00 mg/kg	5.61 ± 1.01	113.50 ± 20.04	36.95 ± 5.78^#^

Note: ^#^ *p* < 0.05 compared with model group.

### Effects of SRC-CDs on the levels of inflammatory cytokines in both plasma and tissues

**FIGURE 7:**
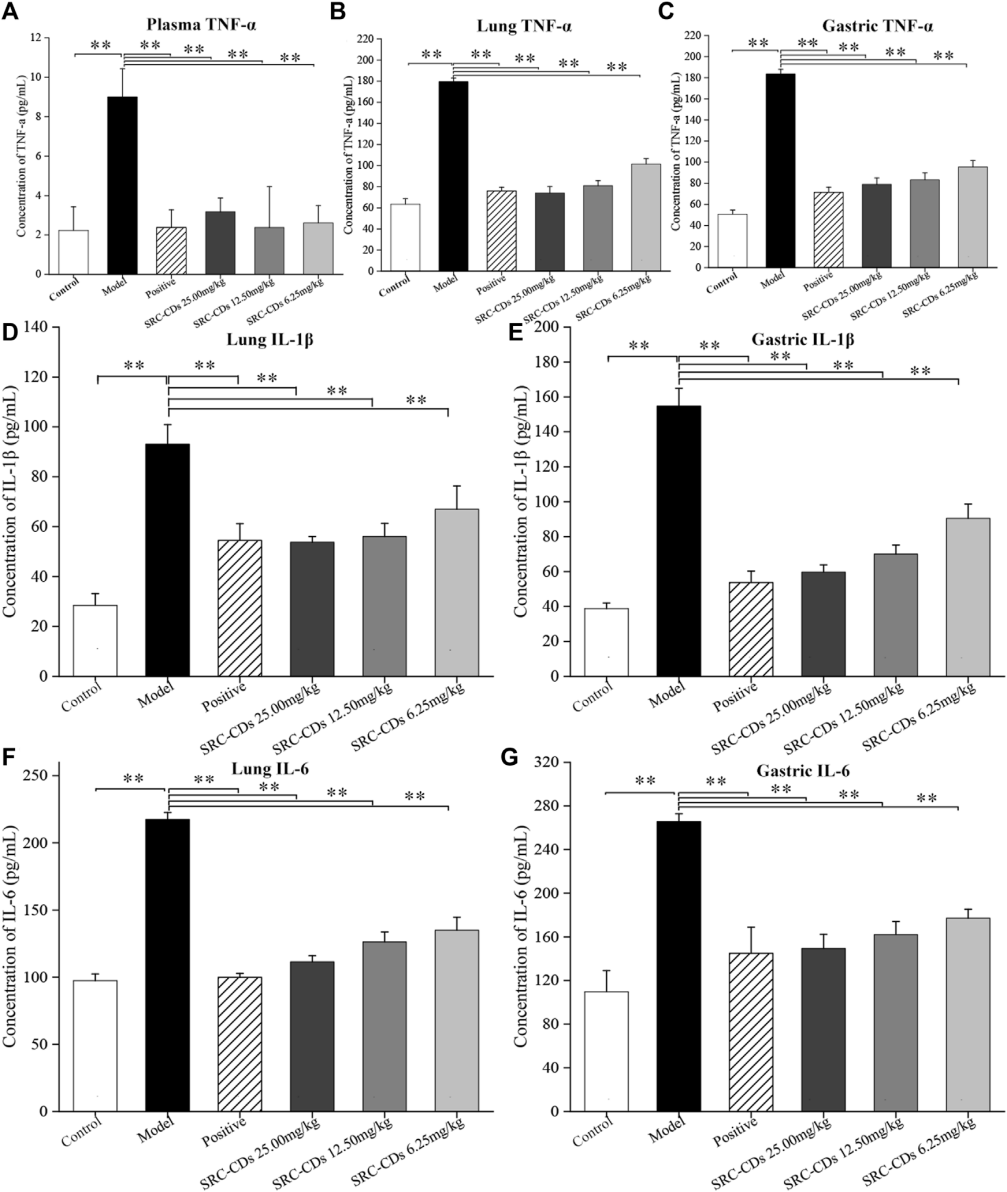
Effects of *Scutellariae Radix Carbonisata—*derived carbon dots (SRC-CDs) on the levels of inflammatory cytokines. The levels of **(A)** TNF-α in plasma, **(B)** TNF-α, **(D)** IL-1β and **(F)** IL-6 in lung tissues, **(C)** TNF-α, **(E)** IL-1β and **(G)** IL-6 in gastric tissues. Sprague Dawley rats were assigned into six groups (*n* = 10): control, model, positive and SRC-CDs at high- (25.00 mg/kg), medium- (12.50 mg/kg) and low (6.25 mg/kg) doses. **p < 0.01 and *p < 0.05 vs. control group or model group.

The concentrations of TNF-α in rat plasma (Figure 7A), lung (Figure 7B) and gastric tissues (Figure 7C) were measured by ELISA method. Compared to control group, the plasma levels of TNF-α was significantly increased in the model group, while positive drug intervention significantly alleviated the elevated trend (*p* < 0.01). Notably, intervention with three doses of SRC-CDs produced a significant reduction (*p* < 0.01) in TNF-α levels, suggesting an anti-inflammation effect of SRC-CDs. In addition, we also examined the levels of IL-6 and IL-1β in both lung (Figures 7D–F) and gastric tissues (Figures 7E, G). The above inflammatory factors were significantly elevated in BHH rats (*p* < 0.01), while the beneficial effects of SRC-CDs at high-, medium- and low-doses on decreasing the IL-6 and IL-1β levels in both lung (Figures 7D, F) and gastric tissues have been demonstrated. A similar phenomenon was observed in animals treated with positive drug. Additionally, since the plasma levels of IL-6 and IL-1β was very low and undetectable, related data were not shown in this study.

### Effects of SRC-CDs on expression of myD88 and NF-κB p65 proteins

**FIGURE 8:**
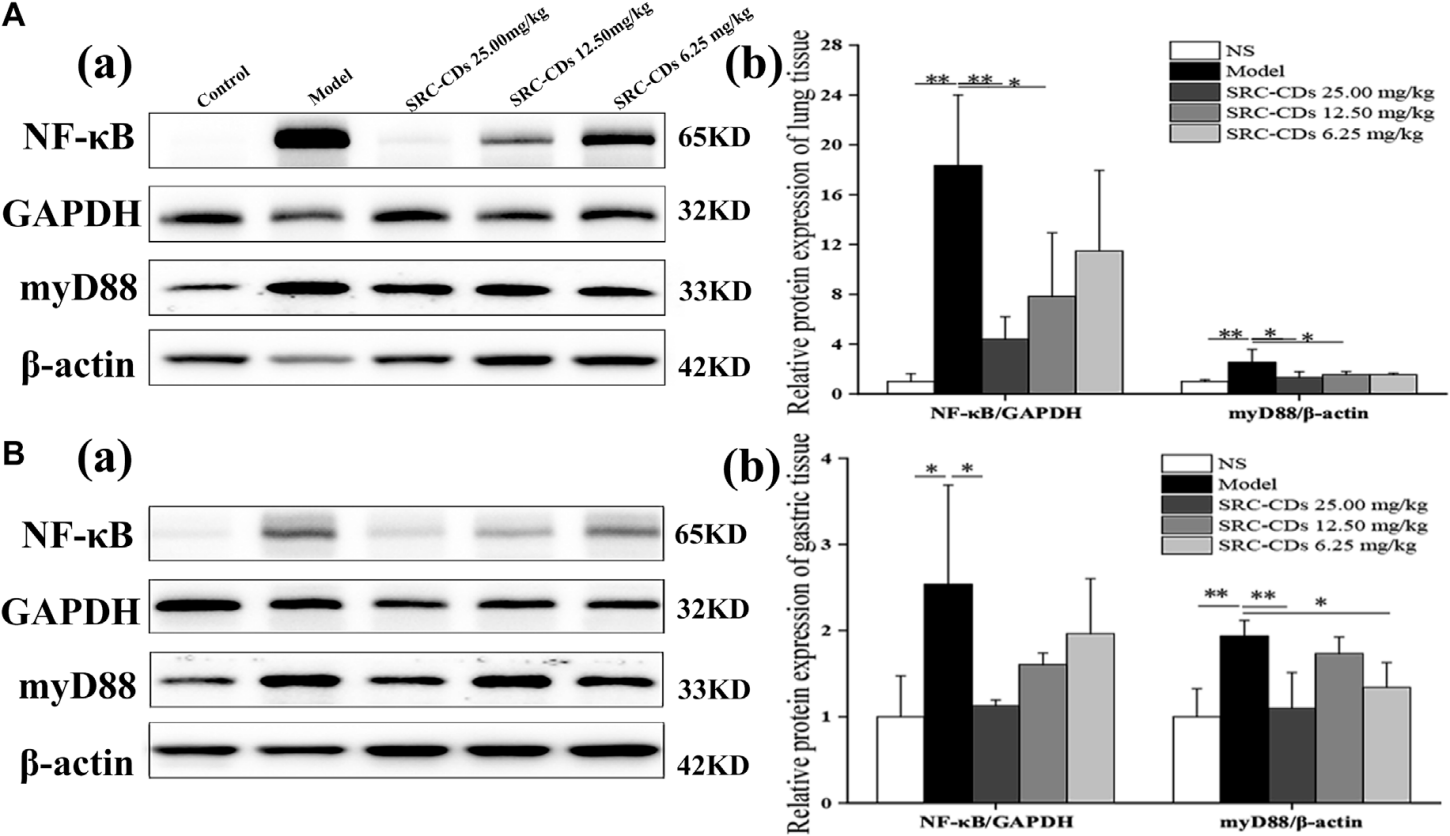
Effects of *Scutellariae Radix Carbonisata—*derived carbon dots (SRC-CDs) on the expression of NF-κB p65 and myD88 proteins in both **(A)** lung and **(B)** gastric tissues. **(A)**, (a) and **(B)**, (a) Representative immunoblot analysis and densitometry. **(A)**, (b) and **(B)**, (b) Quantification of NF-κB p 65 protein and myD88 protein. Sprague Dawley rats were assigned into five groups (*n* = 10): control, model and SRC-CDs at high- (25.00 mg/kg), medium- (12.50 mg/kg) and low (6.25 mg/kg) doses. *p < 0.05 and **p < 0.01 vs. control group or model group.

Compared with the control group, the expression levels of NF-κB p65 (lung tissue: *p* < 0.01; gastric tissue: *p* < 0.05) and myD88 (*p* < 0.01 in both tissues) proteins were significantly increased in both lung (Figure 8A) and gastric tissues (Figure 8B) of model rats, suggesting that the myD88/NF-κB pathways was activated after dry yeast combined with anhydrous ethanol intervention. After treated with high dose of SRC-CDs, lung and gastric expression levels of NF-κB p65 (lung tissue: *p* < 0.01, gastric tissue: *p* < 0.05) and myD88 (lung tissue: *p* < 0.05, gastric tissue: *p* < 0.01) proteins were significantly reduced in BHH animals. Similarly, the expression levels of both proteins were reduced in lung tissues (*p* < 0.05) in BHH rats treated with medium-dose of SRC-CDs. In addition, we found that low-dose SRC-CDs also reversed the elevated gastric protein levels of myD88 that induced by dry yeast combined with absolute ethyl alcohol. NF- κB p65protein expression in the gastric tissues of rats in the medium- and low-dose SRC-CDs groups showed an obvious, although not significant, decrease compared to the model group. Similar observations were made in the lung tissues of model animals. These results indicated that the cooling blood and hemostasis effects of SRC-CDs may be achieved through the inhibition of myD88/NF-κB signalling pathway.

## Discussion

Carbonizing, as the traditional and unique processing technology of traditional Chinese medicine (TCM), has been applied for over 2,000 years, which was first seen in the “Fifty-two prescriptions” of the Han Dynasty. According to TCM theory, carbonized Chinese herbs such as SRC possess new or enhanced hemostatic (or cooling blood and hemostasis) activity compared with crude herbs without carbonizing treatment, which has been validated by long-standing clinical practice and pharmacological experiments (Li et al., 2019; Gao et al., 2021; Liu et al., 2021). In this study, the bleeding time of SRC-treated mice was shorter than that of SR-treated mice, suggesting that SRC possessed an enhanced hemostatic effect than SR. However, despite years of attempts by scholars to investigate the deeper causes of hemostasis of charcoal drugs from the perspectives of hemostatic active substances and mechanisms, this puzzle remains unsolved and has become a major stumbling block for modern research and clinical application of carbonized medicines such as SRC.

In recent years, Qu et al. (Yan et al., 2017; Luo et al., 2018; Sun et al., 2018) proposed that CDs obtained from Chinese medicine by high-temperature carbonization were the hemostatic active components of carbonized medicines, and verified this hypothesis through a series of researches. This novel perspective combining material science with TCM theory is of great guidance to explore the hemostatic and cooling active components in SRC.

Previous reports have shown that SRC exhibited significant hemostatic effects in both mouse tail amputation and liver scratch models, in contrast to its unidentified active ingredient. Therefore, inspired by the above-mentioned observations, the present study prepared the SRC-CDs from the aqueous extracts of SRC, and the following physical and chemical property analysis showed that the SRC-CDs possessed tiny size as well as abundant functional group. Further pharmacodynamic experiment firstly demonstrated that SRC-CDs, not small molecular compounds, was the active components of SRC, which possessed pronounced cooling blood and hemostatic activity. The tiny size and abundant functional groups of as-prepared SRC-CDs exhibited may be the potential factors for their activity on cooling bleeding and hemostasis activity.

According to TCM theory, BHH syndrome is the common clinical hemorrhagic disease, which mainly manifested as hematochezia, metrorrhagia, hematemesis and so on. Although typical bleeding models such as mice tail amputation and liver scratch models (Wu et al., 2013) are commonly used for screening hemostatic drugs, they are less specific when used for studying the activity or mechanism of Chinese medicine with cooling blood and hemostasis. In view of this, a rat model of BHH induced by dry yeast combined with ethanol (Shang et al., 2014; Cheng P. et al., 2019) was constructed and used to investigate the cooling blood and hemostasis activity of SRC-CDs as well as corresponding mechanism in present study.

According to the theory of TCM, blood-heat usually refers to the entry of “exogenous heat” into the blood or “heat” induced by internal damage such as acrimony excitant food, leading to the abnormalities in many blood parameters such as blood counts and coagulation parameters. These abnormalities will manifest as bleeding or blood stasis, which is usually defined as “blood heat hemorrhage” when bleeding symptoms occurred in clinic. To be more in line with the cause of “blood-heat hemorrhage” in TCM, we applied the dry yeast (“exogenous heat”) combined with ethanol (internal damage) as the reagent for establishment blood heat and hemorrhage model based on the previous report. Dry yeast can cause local ulcers at the injection site, acute inflammation, and activated release of endogenous thermogenic substances, leading to pyrexia and chemical bleeding of the body (Gao X. et al., 2013; Gao X. Y. et al., 2013). At the same time, administration with ethanol can destroy gastric mucosa of rats, leading to a severe inflammatory response in the body during modeling combined with dry yeast (Wang et al., 2010). Consequently, rats with blood-heat hemorrhage syndrome developed an elevated rectal temperature, local hemorrhage and severe inflammatory response. In the present study, hemorrhage, erosion, and ulceration were observed in rat gastric tissues after administration of ethanol, indicating significant hemorrhage and inflammatory gastric injury in animals of the model group. Similar observations could be found in the pathological changes of the lung tissue. Combined with the elevated body temperature induced by dry yeast, these manifestations suggested that rat BHH model was successfully established. Notably, all three doses of SRC-CDs intervention greatly reversed pathological changes such as multiple bleeding sites and inflammatory cells infiltration in both gastric and lung tissues. Similar trends were observed in improving elevated rectal temperature and inflammatory cytokine levels in plasma, lung, and gastric tissues, demonstrating that SRC-CDs exerted cooling blood and hemostasis effects through hemostasis and anti-inflammation.

Further studies revealed that SRC-CDs exerted hemostatic efficacy by activating endogenous coagulation pathways and the FIB system, mainly manifested as effectively alleviating elevated APTT and FIB induced by modelling agents. Additionally, the anti-inflammation activity of SRC-CDs was significantly associated with reduced levels of TNF-α, IL-6, and IL-1β through inhibiting myD88/NF-κB signaling pathway. The above results provided some explanation for the mechanism of SRC-CDs in cooling blood and hemostasis.

Carbon dots (CDs), as a newcomer of carbon-based materials, are “zero-dimensional” carbon-based nanomaterials with unique advantages (Zhang et al., 2014; Chellasamy et al., 2021) of tiny size, abundant functional groups and hypotoxicity. Previous studies have indicated that CDs obtained from some other carbonized herbs such as Junci Medulla Carbonisata (Cheng J. et al., 2019) and Phellodendri Cortex Carbonisata (Liu et al., 2018) also exerted hemostatic activity, but most of them were evaluated by classical mice tail amputation and liver scratch models. In terms of the cooling blood and hemostasis effects of CDs-derived carbonized herbs, the present study is the first attempt and demonstration. Since the mechanisms of CDs exhibited certain differences, it is of great interest to reveal the common influencing factors and differences in regulatory mechanisms in the hemostatic efficacy of CDs derived from different charcoal drugs, which have not yet been explored and requires in-depth investigation.

## Conclusion

In this study, we demonstrated for the first time that SRC-CDs with tiny size and abundant functional groups were the blood cooling and hemostatic active component of SRC. The potential mechanism was partly through the inhibition of myD88/NF-κB cell signaling pathway, activation of fibrin system and endogenous coagulation pathway to exert anti-inflammatory and hemostatic effects. Our findings not only provide evidence base for further elucidation of the cool blood and hemostasis mechanism of SRC-CDs, but also provide a beneficial and novel methodological reference for research on the active ingredients of TCM.

## Data Availability

The original contributions presented in the study are included in the article/Supplementary Material, further inquiries can be directed to the corresponding authors.
