# Supplementary figures and images for: Protective effects of Scutellariae Radix Carbonisata-derived carbon dots on blood-heat and hemorrhage rats
Source: Front Pharmacol. 2023 Aug 10;14:1118550. doi: 10.3389/fphar.2023.1118550 (PMC10450154; doi:10.3389/fphar.2023.1118550)

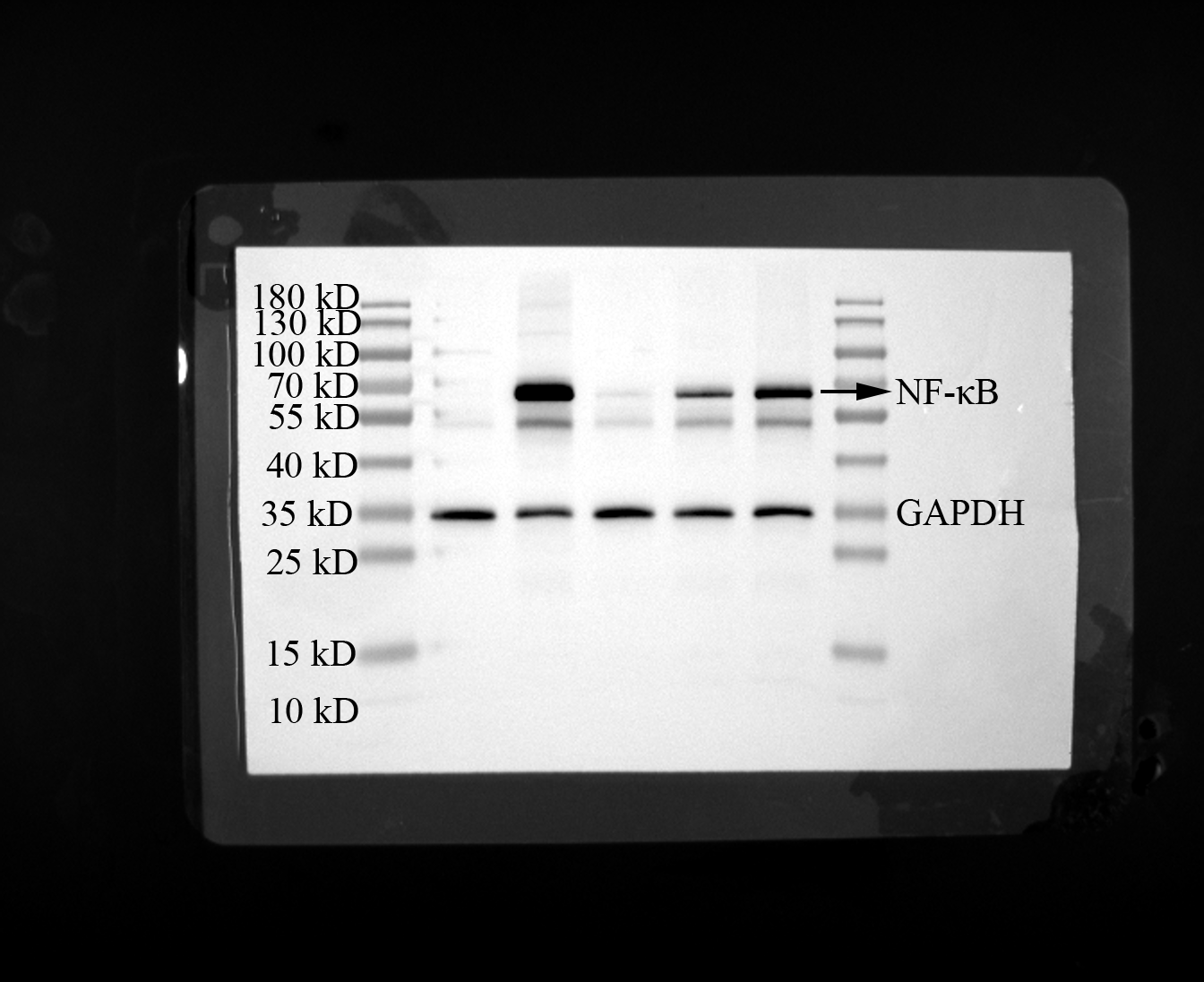

Supplement: Supplementary file 1 [file DataSheet1.ZIP › Supplementary material/Fig 8 A(a)-NF-κB-GAPDH-Merge.Tif]

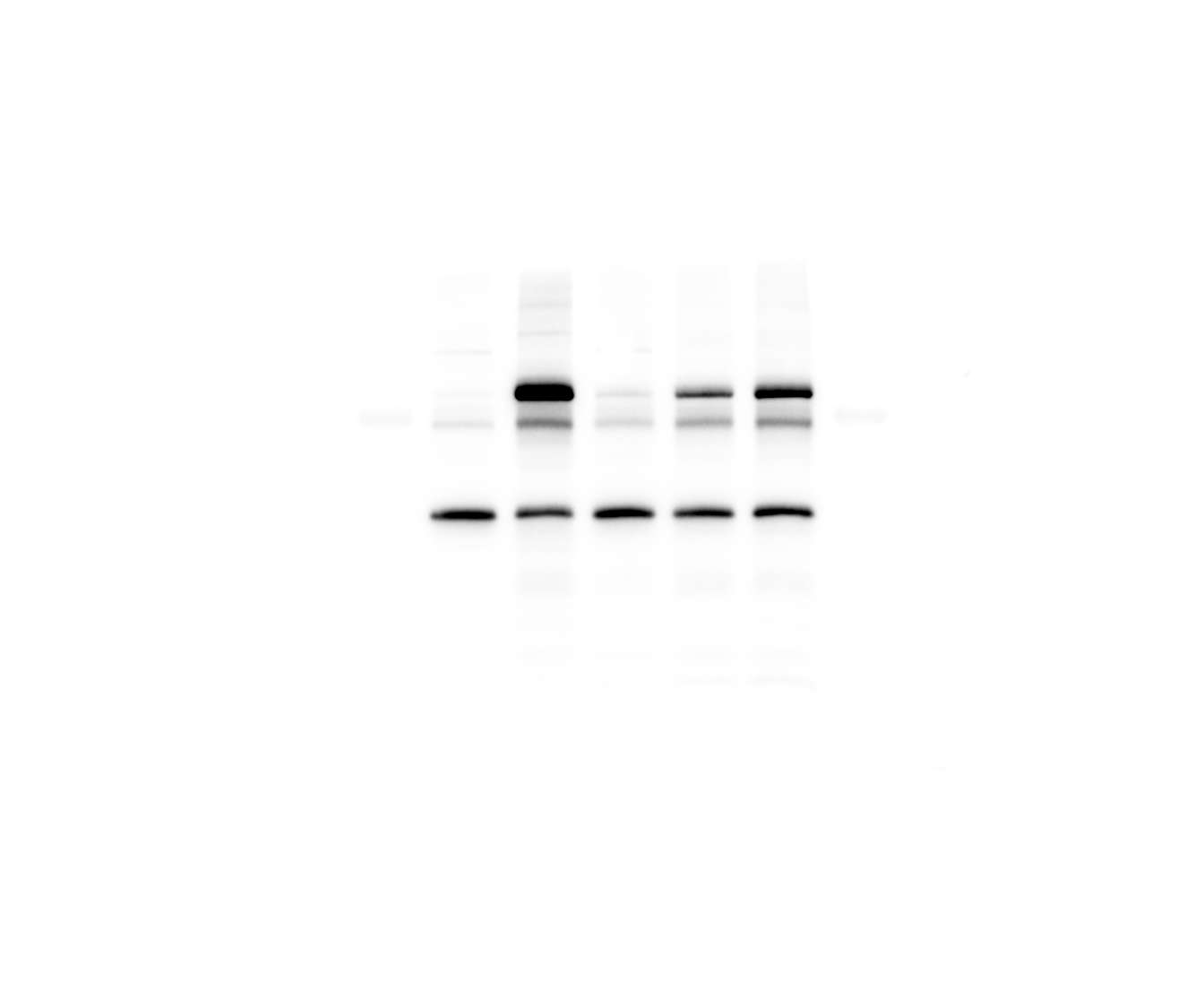

Supplement: Supplementary file 1 [file DataSheet1.ZIP › Supplementary material/Fig 8 A(a)-NF-κB-GAPDH.Tif]

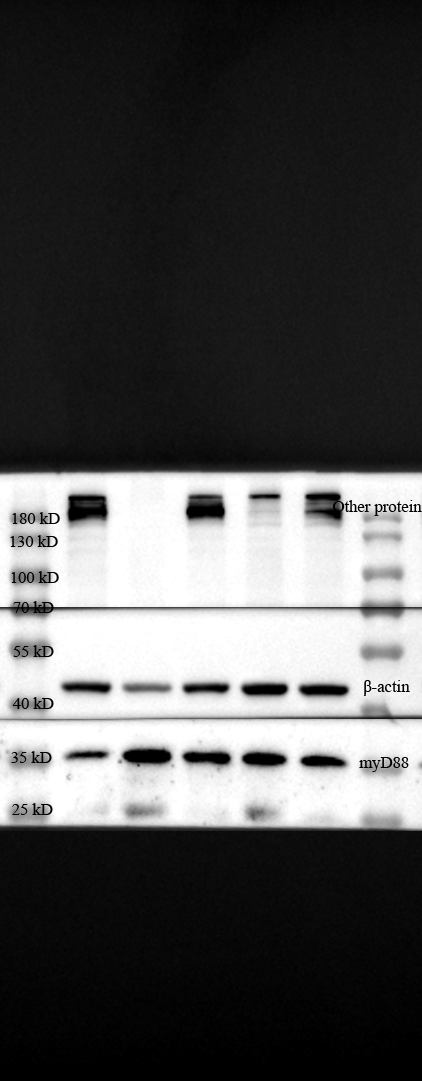

Supplement: Supplementary file 1 [file DataSheet1.ZIP › Supplementary material/Fig 8 A(b) myd88 and β-actin -merge .tif]

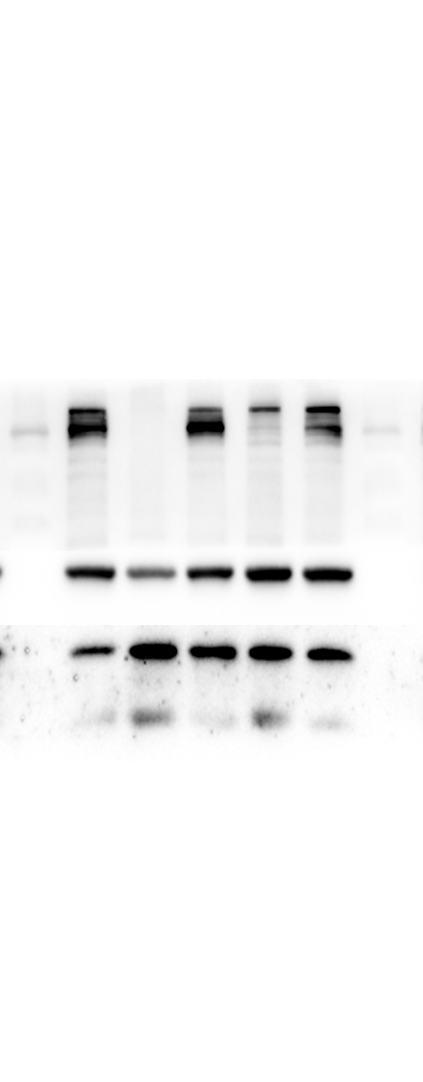

Supplement: Supplementary file 1 [file DataSheet1.ZIP › Supplementary material/Fig 8 A(b) myd88 and β-actin .Tif]

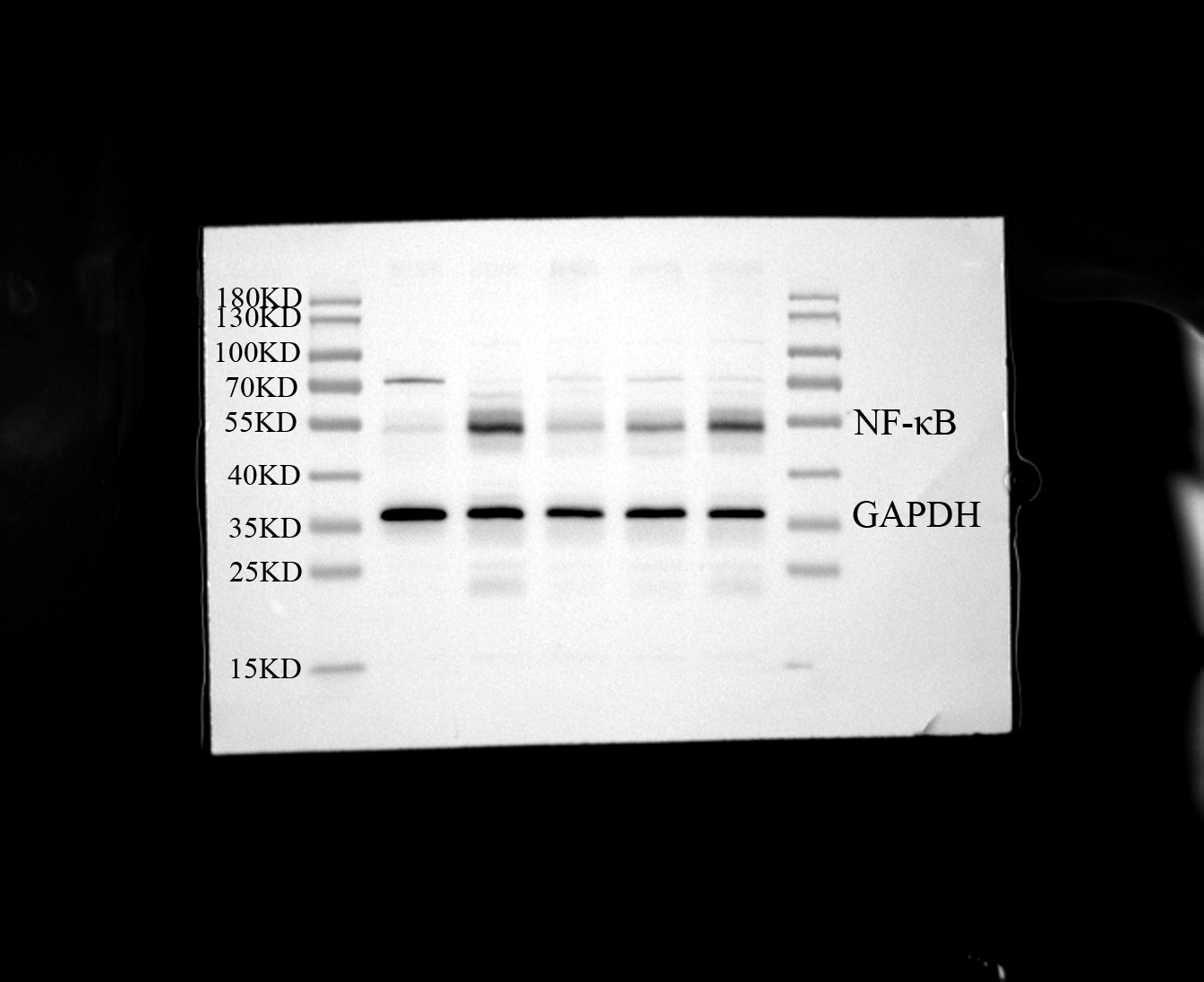

Supplement: Supplementary file 1 [file DataSheet1.ZIP › Supplementary material/Fig 8 B(a)-NF-κB-GAPDH-Merge.Tif]

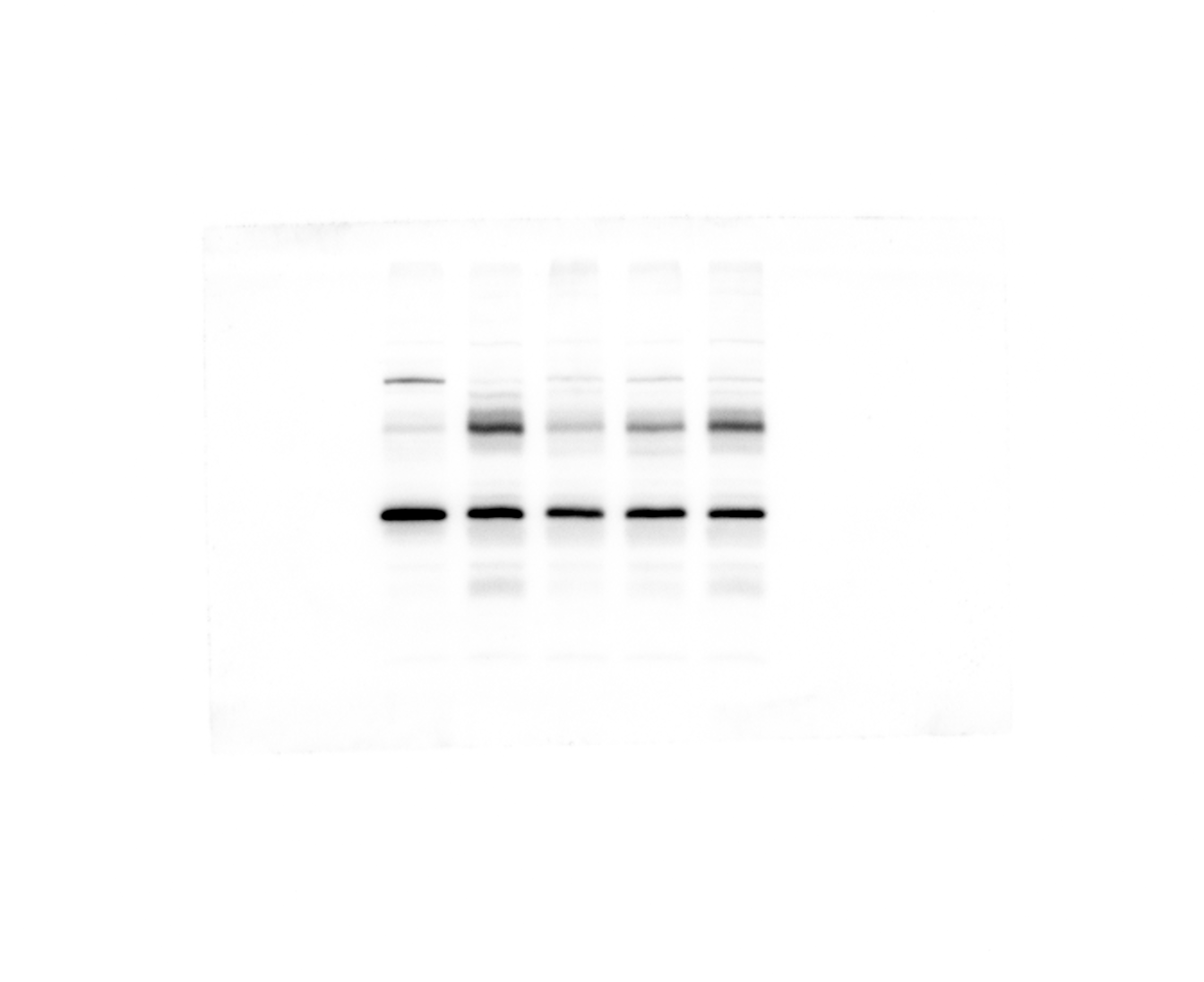

Supplement: Supplementary file 1 [file DataSheet1.ZIP › Supplementary material/Fig 8 B(a)-NF-κB-GAPDH.Tif]

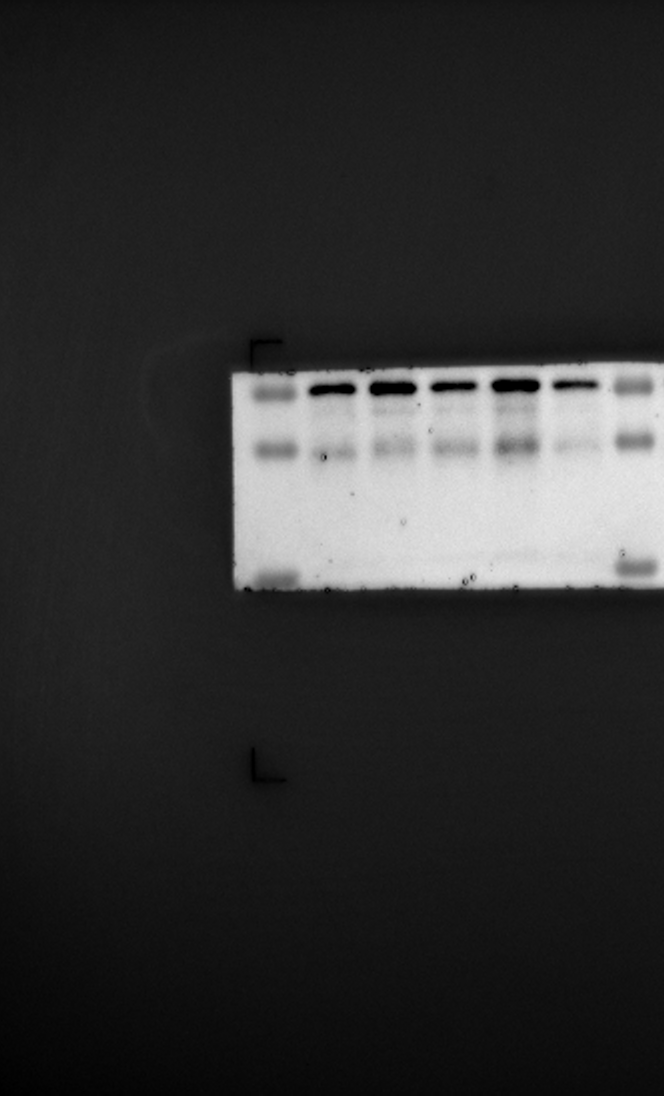

Supplement: Supplementary file 1 [file DataSheet1.ZIP › Supplementary material/Fig 8 B(b) myd88 -merge.Tif]

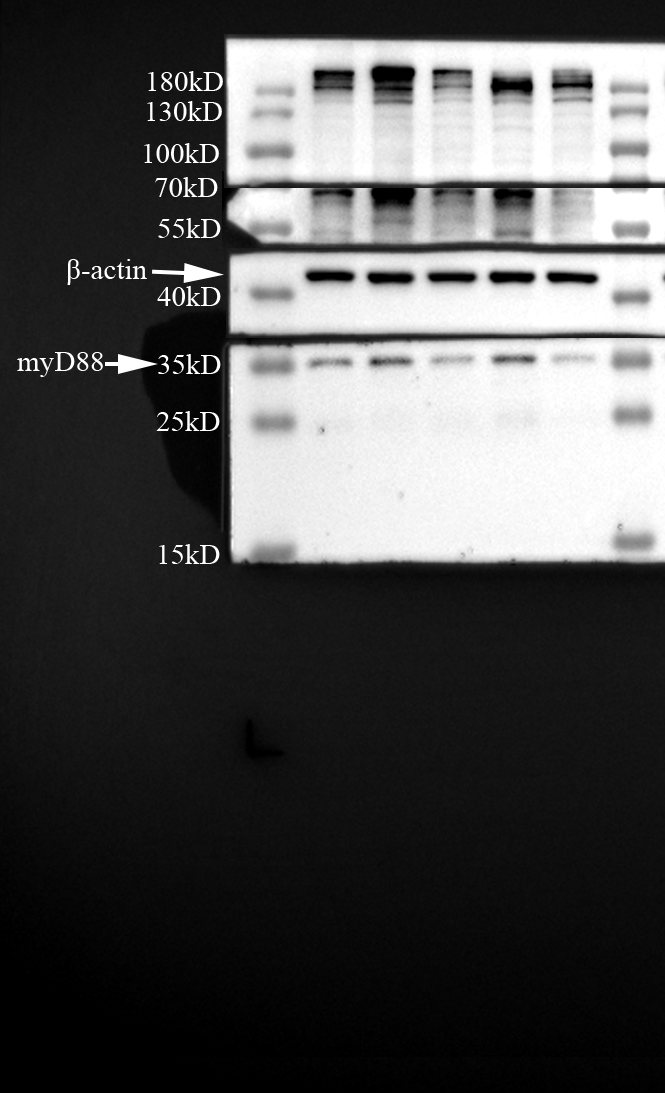

Supplement: Supplementary file 1 [file DataSheet1.ZIP › Supplementary material/Fig 8 B(b) myd88 and β-actin -entire gel.tif]

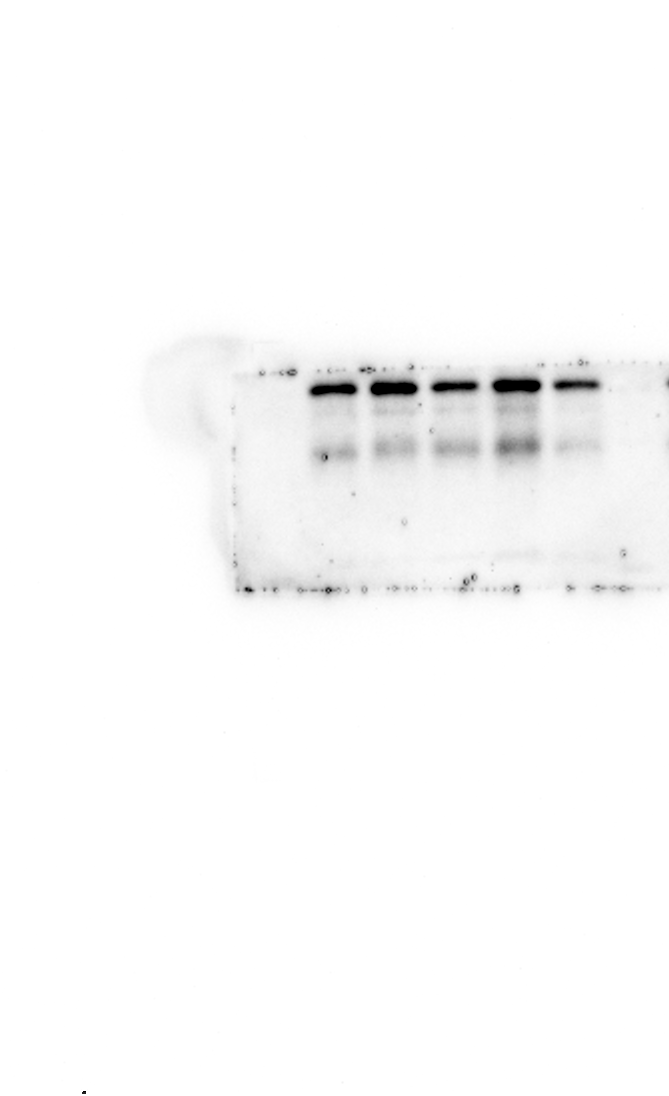

Supplement: Supplementary file 1 [file DataSheet1.ZIP › Supplementary material/Fig 8 B(b) myd88.Tif]
